# *In Vitro* Antimicrobial Evaluation of Silver Nanoparticles in Lyotropic Liquid Crystals for Cutaneous Wound Treatment

**DOI:** 10.1021/acsomega.5c09016

**Published:** 2026-01-31

**Authors:** Franciele Garcia Baveloni, Marco Antônio Utrera Martines, Bruna Almeida Furquim de Camargo, Marcela Tavares Luiz, Melina Borges Teixeira Zanatta, Isabella Carvalho Pereira da Silva, Hernane da Silva Barud, Guillermo R. Castro, Amauri Antônio Menegário, Taís Maria Bauab, Andréia Bagliotti Meneguin, Marlus Chorilli

**Affiliations:** † Department of Drugs and Medicines, 28108 São Paulo State University (UNESP), Araraquara, São Paulo 14800-901, Brazil; ‡ Institute of Chemistry, Federal University of Mato Grosso Do Sul (UFMS), Campo Grande, Mato Grosso Do Sul 79074-460, Brazil; § Center for Environmental Studies, 123238 São Paulo State University (UNESP), Rio Claro, São Paulo 13506-900, Brazil; ∥ Laboratory of Biopolymers and Biomaterials, University of Araraquara (UNIARA), Araraquara, São Paulo 14801-320, Brazil; ⊥ Nanomedicine Research Unit, Center for Natural and Human Sciences, Federal University of ABC (UFABC), Santo André, São Paulo 09210-580, Brazil

## Abstract

Complex wounds, burns, and diabetic complications increase patients’ susceptibility to bacterial infections, which frequently show resistance to standard treatments. This investigation focused on evaluating the antimicrobial potential of lyotropic liquid crystal (LC) formulations loaded with silver nanoparticles (AgNPs) against *Staphylococcus aureus*, *Pseudomonas aeruginosa*, and *Escherichia coli* strains. The liquid crystals (LCs) were prepared by incorporating an oily phase (PPG-5-CETETH-20 and oleic acid) into an aqueous phase containing the AgNP nanosuspension. LC-AgNPs formulations, containing AgNPs concentrations between 4.68 μg/mL and 14.04 μg/mL, were examined using polarized light microscopy (PLM), which identified hexagonal, Maltese-cross/lamellar, and dark-field mesophases. The ratio between the aqueous and oily phases directly affected the mechanical, bioadhesive, and rheological properties of the LCs. Over a 24 h period, the formulations released between 1% and 70% of the AgNPs, showing proven efficacy against the tested bacterial strains. The structural features of LC formulations significantly influenced their biocompatibility in L-929 fibroblast cells. The findings demonstrate that LC systems are promising controlled-release platforms for AgNPs, combining antimicrobial activity and biocompatibility for the treatment of bacterial skin infections.

## Graphical abstract

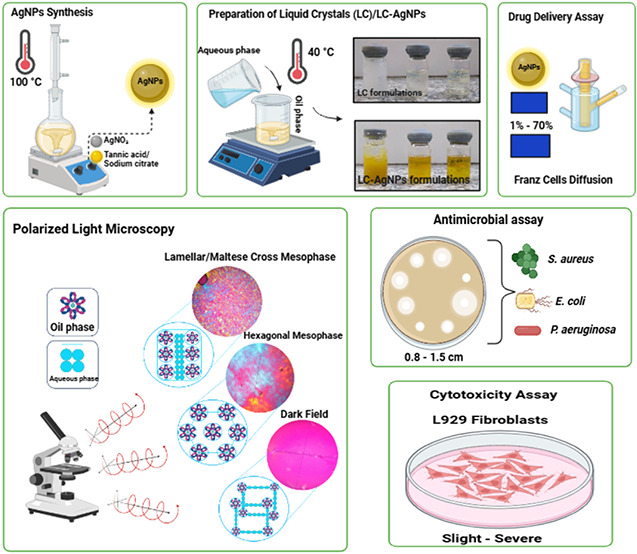

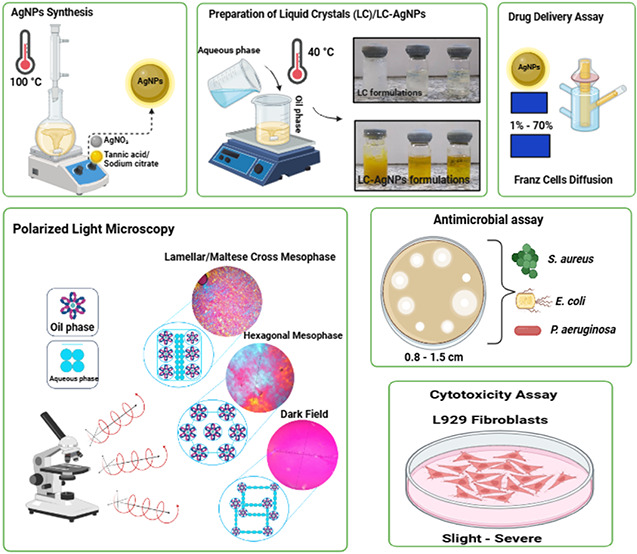

## 1 Introduction

Skin lesions caused by burns, metabolic diseases such as diabetes, and complex wounds that fail to heal due to pathogenic bacteria represent a critical worldwide health concern.^1^ In 2010, skin diseases ranked as the fourth leading cause of quality-of-life impairment due to disability.^2^ Beyond contributing to prolonged hospitalizations, these conditions often result in disfigurement and permanent disabilities, imposing a substantial socioeconomic burden, including high treatment costs and loss of productivity.^3^

Skin wounds are particularly susceptible to infections due to the loss of skin integrity and the immunosuppression resulting from trauma. The compromised skin barrier, which serves as the main barrier preventing pathogen entry, makes these wounds highly vulnerable to bacterial invasion, further complicating the healing process.^4^ Among the primary pathogens, *S. aureus*—particularly methicillin-resistant strains (MRSA)—is frequently associated with hospital-acquired infections.^5^ Additionally, *P. aeruginosa* predominates in burn wounds due to its ability to colonize moist surfaces and form biofilms, which complicates treatment.^6^ Severe secondary infections, such as those caused by *E. coli* in immunocompromised patients, further exacerbate the clinical condition.^7^ These pathogens contribute to complications and high mortality rates in burn patients, highlighting the urgent need for novel therapeutic approaches.^8^

Silver, widely used in pharmaceutical formulations such as silver sulfadiazine and AgNP creams, is a well-established antimicrobial agent for burn treatment. Its potent antimicrobial properties help prevent infections in injured areas while promoting wound healing.^9^ Furthermore, silver has demonstrated effectiveness in treating pressure ulcers, chronic wounds, and diabetic lesions, making it a valuable option for combating bacterial strains.^10^

AgNPs offer several advantages over silver sulfadiazine in burn treatment. While silver sulfadiazine is known for its antimicrobial properties, AgNPs exhibit a broader antimicrobial spectrum and demonstrate efficacy at lower concentrations, thereby reducing the risk of toxicity.^11^ Additionally, due to their nanoscale size, AgNPs possess enhanced penetration capabilities and can be incorporated into sustained-release systems, including lyotropic LCs, allowing for sustained and targeted action.^12,13^

LCs are formed by adding solvents to amphiphilic molecules under specific physicochemical conditions, resulting in structures that combine characteristics of both liquid and solid crystals.^14^ These systems consist of molecules with distinct polar and nonpolar regions, enabling large-scale molecular organization. Due to their exceptional structural properties—including high drug-loading capacity, controlled release, and flexibility—LCs have gained increasing interest as innovative systems to enhance skin permeability.^15^ Moreover, their strong bioadhesion and structural similarity to biological membranes make them particularly effective for topical applications, optimizing both drug retention and skin permeation.^16^

Although AgNPs have been shown to be dispersible in nematic and lamellar LC systems,^17,18^ few or no studies have investigated the incorporation of AgNPs into lyotropic LC as a controlled-release platform for the treatment of cutaneous wounds.^19,20^

Our study fills this gap by developing a lyotropic liquid LC system containing AgNPs and evaluating its antimicrobial activity and wound-healing potential in skin lesions. This formulation offers several advantages, including sustained release of the active agent, enhanced adhesion to the skin tissue, reduced toxicity, and feasibility for practical topical application. Thus, the proposed approach aims not only to minimize possible adverse effects but also to enhance the antimicrobial efficacy against bacterial strains commonly associated with cutaneous wounds, representing a promising alternative for the treatment of such lesions.

## 2 Experimental Section

### 2.1 Materials

Silver nitrate (AgNO_3_, analytical grade), tannic acid (analytical grade), and sodium citrate (analytical grade) were obtained from Sigma-Aldrich (St. Louis, MO, USA). Oleic acid (Synth, Brazil) was used as the oil phase. The surfactant PPG-5-CETETH-20 (Procetyl AWS, Croda Pharma, UK) was obtained from a commercial supplier. Ultrapure water (resistivity 18.2 MΩ·cm) was used in all preparations. Cellulose acetate membranes (molecular weight cutoff 12–14 kDa, 1.77 cm^2^) were purchased from Millipore (or equivalent). The components of the simulated exudate fluid (SEF)—sodium and calcium salts—were analytical grade and obtained from Sigma-Aldrich (or equivalent). Mueller Hinton agar and reagents for microbiological assays were supplied by Oxoid/Thermo Fisher Scientific (or equivalent).

Pig ear skin used for *ex vivo* assays was obtained from a local abattoir. Cell culture reagents, including Dulbecco’s Modified Eagle Medium (DMEM), fetal bovine serum (FBS), and antibiotics, were purchased from Gibco/Thermo Fisher Scientific (or equivalent). Triton X-100 (positive control for cytotoxicity) and neutral red were obtained from Sigma-Aldrich.

The instruments and equipment used included: an Olympus BX41 polarized light microscope equipped with a QColor3 camera (Olympus America Inc.); a TA.XTPlus texture analyzer (Stable Micro Systems, UK); an AR2000 controlled-stress rheometer (TA Instruments, New Castle, DE, USA) with cone–plate geometry (40 mm diameter); Franz diffusion cells (Microette, Hanson Research, Chatsworth, CA, USA); a Sorvall centrifuge (model as described in the Methods section); an Ultra-Turrax homogenizer (IKA, Germany); an ultrasonic bath; and an iCAP Qnova Series ICP-MS system (Thermo Fisher Scientific, Waltham, MA, USA). All other reagents and materials were of analytical grade and used as received.

### 2.2 Synthesis of AgNPs

The synthesis and characterization procedures for AgNPs followed the methodology previously reported by Baveloni et al. (2024).^9^ In summary, AgNPs were produced at a final concentration of 117 μg/mL using aqueous mixtures containing tannic acid (0.025 mM), sodium citrate (5 mM), and silver nitrate (AgNO_3_, 25 mM), with the reaction carried out under boiling conditions at 100 °C. Comprehensive physicochemical analyses, including UV–Vis spectroscopy, particle size evaluation by TEM and DLS, and zeta potential determination, were described in detail in the referenced study.

### 2.3 Preparation of LC Formulations

The development of the LC system was based on the adapted formulation by Calixto et al. (2018), using water as the aqueous phase, oleic acid (Synth) as the oil phase, and PPG-5-CETETH-20 as the surfactant (refer to the concentrations in Table 1). The mixture was maintained under magnetic agitation at 40 °C until a homogeneous system was formed.^21^

**Table 1:** Classification of Liquid-Crystalline Formulations^a^

	Concentrations (%)					
Formulations	Water/AgNPs	PPG-5-CETETH-20	Oleic acid	PLM	PLM LCs-AgNPs	Stability LC-AgNPs
**LC**_**1**_	20	70	10	Maltese-cross/Hexagonal	Dark Field	Oxidation
**LC**_**2**_	30	60	10	Maltese-cross/Hexagonal	Maltese-cross/Hexagonal	Oxidation
**LC**_**3**_	40	50	10	Dark Field	Maltese-cross/Hexagonal	Oxidation
**LC**_**4**_	50	40	10	Hexagonal	Maltese-cross/Hexagonal	Oxidation
**LC**_**5**_	60	30	10	Hexagonal	Hexagonal	Stable
**LC**_**6**_	20	60	20	Maltese-cross/Hexagonal	Maltese-cross/Hexagonal	Stable
**LC**_**7**_	30	50	20	Hexagonal	Hexagonal	Oxidation
**LC**_**8**_	40	40	20	Maltese-cross/Hexagonal	Maltese-cross/Hexagonal	Oxidation
**LC**_**9**_	50	30	20	Hexagonal	Hexagonal	Oxidation
**LC**_**10**_	20	50	30	Dark Field	Maltese-cross/Hexagonal	Stable
**LC**_**11**_	40	30	30	Hexagonal	Hexagonal	Oxidation

a Components of the liquid-crystalline formulations. Aqueous phase: Water and AgNPs. Surfactant: PPG-5-CETETH-20. Oil phase: Oleic acid. Polarized light microscopy (PLM): Dark field, maltese-cross, and hexagonal structures. Stability of LC-AgNPs: Stable and oxidation.

#### 2.3.1 Preparation of LC Formulations Containing AgNPs

The LC system containing AgNPs (LC-AgNPs) was prepared using AgNPs derived from prior studies. Specifically, 117 μg/mL AgNPs were utilized for the preparation of the liquid crystalline formulations, following the same method described in Section 2.3.

### 2.4 PLM Analysis of LC and LC-AgNPs Formulations

PLM was employed to evaluate the mesophase organization of the LC and LC-AgNP systems prepared as described in Sections 2.3 and 2.3.1. For this analysis, an aliquot of each selected formulation was transferred onto a glass slide, carefully covered with a coverslip, and examined at 20× magnification. The observations and image acquisition were carried out using an Olympus BX41 optical microscope equipped with a QColor3 digital camera from Olympus America Inc.

### 2.5 Evaluation of the Stability of LC-AgNPs Formulations

The formulations were stored at 5 and 25 °C for visual macroscopic evaluation at 48 h after their preparation. LC-AgNPs formulations that maintained their yellow coloration during the observation period are considered stable and they were selected for subsequent studies.

### 2.6 Texture Profile Analysis

Texture profile analysis (TPA) was conducted using a TA.XT Plus texture analyzer from Stable Micro Systems, United Kingdom. Samples with a mass of 7.0 g were placed in 10 mL conical centrifuge tubes and centrifuged in a Sorvall TC 6 system to eliminate entrapped air. After centrifugation, the samples were allowed to rest for 24 h before analysis. Measurements were performed using a 10 mm cylindrical probe. The probe approached the sample at a speed of 1 mm per second and compressed it to a depth of 10 mm. After retraction at 0.5 mm per second and a 5 s interval, a second compression cycle was applied. Force–time curves were recorded, and the parameters hardness, compressibility, adhesiveness, and cohesiveness were calculated. All tests were performed in triplicate at 25 °C ± 0.5 °C.

### 2.7 *In Vitro* Bioadhesion Analysis

The bioadhesive behavior of the formulations was assessed under *in vitro* conditions using a TA.XT Plus texture analyzer configured for adhesion testing. The evaluation was based on the maximum force required for detachment and the corresponding work of adhesion between the formulation and the skin substrate.

Porcine ear skin was prepared by dermatoming to a thickness of 500 μm with a Nouvag TCM 300 device and then equilibrated in a 0.9% sodium chloride solution for 15 min. After hydration, the skin sections were fixed onto a cylindrical analytical probe. Formulations were placed in 10 mL centrifuge tubes and positioned below the probe prior to testing.

During the assay, the probe was lowered at a constant rate of 1 mm per second until contact was established, which was maintained for 60 s. Detachment was then performed by raising the probe at 0.05 mm per second, and force–time profiles were recorded. All experiments were conducted in triplicate at a controlled temperature of 32 °C with a variation of ±0.5 °C.

### 2.8 Rheological Analysis

The rheological behavior of LC and LC-AgNP formulations was evaluated using a controlled-stress AR2000 rheometer from TA Instruments, New Castle, DE, United States. A cone–plate geometry with a diameter of 40 mm and a gap of approximately 50 μm was employed. To minimize shear-related effects, the formulations were gently distributed onto the lower plate and allowed to stabilize for about 3 min prior to analysis. Measurements were carried out with 3.0 g of sample under two temperature conditions, namely 25.0 °C ± 0.5 °C and 32 °C ± 0.5 °C, corresponding to ambient and skin temperatures, respectively, and performed in triplicate.

#### 2.8.1 Evaluation of Flow Properties

Flow behavior was assessed using a controlled shear rate protocol in which the shear rate was varied from 0.01 to 100 s^–1^ and subsequently returned to its initial value. The analysis was conducted over a 120 s period, with a 10 s interval between the increasing and decreasing shear curves. The consistency (κ) and flow (η) indices were obtained using the power law model presented in eq (1), providing a quantitative description of the flow properties.^22,23^

#### 2.8.2 Oscillatory Rheological Analysis

Oscillatory measurements began with a stress sweep performed at 1 Hz, covering a stress range from 0.1 to 10.0 Pa, to define the linear viscoelastic region (LVR) of the samples. In sequence, a frequency sweep was performed under a constant shear stress of 1.0 Pa, covering a frequency range from 0.1 to 10.0 Hz—previously established within the LVR for all tested formulations. During the analysis, the storage modulus (*G*′) and loss modulus (*G*″) were recorded, enabling the evaluation of the viscoelastic behavior of the samples. At low-frequency ranges, changes in the storage modulus *G*′ were evaluated by plotting *G*′ against the angular frequency ω on a logarithmic scale, in accordance with the power law model.^24^

### 2.9 *In Vitro* Release Analysis

To evaluate the release profile, a 200 μL aliquot of the AgNP nanosuspension at a concentration of 23.4 μg/mL and 200 mg samples of LC_5_-AgNPs at 14.04 μg/mL, LC_6_-AgNPs, and LC_10_-AgNPs at 4.68 μg/mL were placed into the donor compartment of Franz diffusion cells with a capacity of 7.0 mL using Microette equipment (Hanson, Chatsworth, USA). Simulated exudate fluid (FES), containing 142 mmol/L sodium ions and 2.5 mmol/L calcium ions at pH 4.0, was employed as the receptor medium. The receptor solution was maintained under sink conditions with continuous stirring at 300 rpm and a controlled temperature of 32.0 °C ± 0.5 °C. A synthetic cellulose acetate membrane with an effective area of 1.77 cm^2^ and a molecular weight cutoff of 12–14 kDa was conditioned in the receptor medium for 16 h prior to use. Aliquots of 2.0 mL were automatically withdrawn at 0.5, 1, 2, 4, 6, 8, 10, 12, and 24 h using a micropipette system (Hanson 0700–1251) and immediately replaced with an equivalent volume of fresh dissolution medium. The tubes were then immersed in an ultrasound bath for 30 min. Inductively Coupled Plasma Mass Spectrometry (ICP-MS) was used to measure AgNPs in the supernatant (method described in Section 2.11). The assay was performed in quintuplicate.

### 2.10 *Ex Vivo* Release Analysis

*Ex vivo* permeation and retention of AgNP nanosuspension at 23.4 μg/mL and LC-AgNP formulations were investigated using Franz diffusion cells.^25^ Dermatomed porcine ear skin with a thickness of 500 μm was prepared, stored frozen, and equilibrated in phosphate-buffered saline at pH 7.4 prior to use. The skin was mounted in the diffusion system with the stratum corneum facing the donor compartment. The experiments utilized a Franz cell diffusion system (Hanson Vertical Diffusion Cell; Microette Plus, Hanson Research, CA). For the assay, 200 mg of each formulation—including LC_5_-AgNPs at 14.04 μg/mL, LC_6_-AgNPs, and LC_10_-AgNPs at 4.68 μg/mL—were applied onto the skin, which was maintained in contact with simulated exudate fluid (FES) at pH 4.0. Samples were withdrawn from the receptor compartment at predetermined time points of 0.5, 1, 2, 4, 8, 12, and 24 h. At the end of the experiment, the pig skin samples were gently wiped with soft paper and cut into small pieces using scissors. Skin fragments were transferred to Falcon BD tubes containing 4.0 mL of methanol and homogenized using an Ultra-Turrax at 10,000 rpm for 2 min. The Ag concentrations in the supernatant were quantified by ICP-MS, with all assays performed in five replicates.

### 2.11 Quantification of Ag by ICP-MS Analysis

Silver released from AgNPs in the receptor medium from the in vitro and ex vivo assays was quantified by ICP-MS using an iCAP Qnova Series system (Thermo Fisher Scientific, USA). Samples were prediluted in 2.0% (v/v) HNO_3_. Calibration curves were prepared using Ag^+^ standards ranging from 0.5 to 50.0 μg/L, with measurements performed at *m*/*z* 107. Yttrium and indium were used as internal standards to correct signal fluctuations. The limit of detection, calculated as 3σ from ten blank measurements, was 0.012 μg/L.

### 2.12 Antimicrobial Assays

The antimicrobial activity of the LC formulations was evaluated using a disc diffusion assay adapted from ISO 20776–1:2006 and CLSI guidelines.^26^ Mueller–Hinton agar plates were inoculated with standardized suspensions of *S. aureus* (ATCC 25923), *P. aeruginosa* (ATCC 27853), and *E. coli* (ATCC 25922) adjusted to 0.5 McFarland. Sterile filter paper discs containing the LC formulations were placed on the agar surface, and the plates were incubated at 37 °C for 24 h. Inhibition zones were measured using a caliper, with ampicillin at 20 μg/L used as a positive control. All tests were performed in triplicate and analyzed by one-way ANOVA followed by Tukey’s post hoc test.

### 2.13 Evaluation of the Cytotoxicity of LC Formulations

The cytotoxicity of the LC formulations was assessed by a qualitative agar overlay assay in accordance with ISO 10993-5 guidelines.^27^ L-929 fibroblasts were cultured in DMEM supplemented with 10% FBS under standard conditions until a confluent monolayer was formed in 6-well plates. After agar overlay containing neutral red was applied, sterile filter paper discs loaded with the formulations were placed onto the agar surface. Cytotoxicity was evaluated after 24 h by measuring the inhibition halo diameter. DMEM and Triton X-100 were used as negative and positive controls, respectively. All experiments were performed in triplicate and analyzed using one-way ANOVA followed by Tukey’s post hoc test.

## 3 Results and Discussion

### 3.1 Characterization of LC and LC-AgNPs Formulations Using PLM

LC formulations were obtained based on a ternary phase diagram reported by Calixto et al. (2018).^21^ Table 1 presents the concentrations of the components used and the corresponding mesophases observed. The balance between the polar and nonpolar components was essential to drive the self-assembly process leading to the formation of ordered mesophases. However, the presence of unsaturated bonds in the oleic acid structure may also favor moderate oxidative processes when in contact with metallic nanoparticles, particularly under heating and oxygen exposure conditions.^28–31^ These interactions can alter the surface chemistry of AgNPs—potentially leading to the formation of silver carboxylates or oxide layers—and influence their optical properties and stability within the LC matrix.^32^ This behavior helps explain the color changes observed upon AgNPs incorporation, as illustrated in Figure 1.

**Figure 1:**
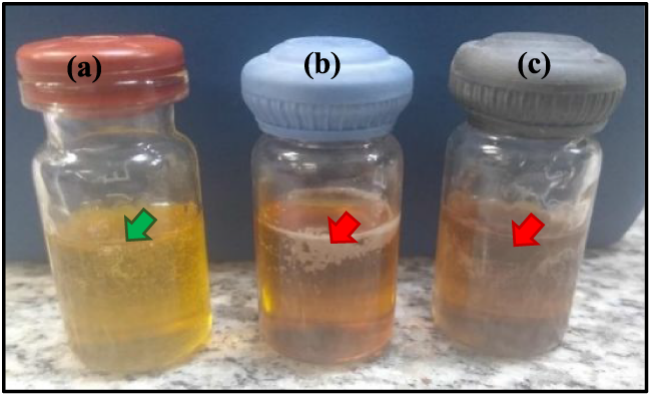
Macroscopic evaluation of Liquid-Crystalline (LC) formulations: (a) the green arrow represents the stable LC formulation (yellow color), and (b) and (c) the red arrow indicates the unstable formulations (dark color).

After AgNP incorporation, LC_5_, LC_6_, and LC_10_ were selected due to their enhanced nanoparticle stability, with their composition and macro- and microscopic characteristics summarized in Table 1.

Additionally, Table 1 and Figure 2 highlight structural differences among the LC formulations, showcasing their respective mesophases. These variations are primarily attributed to the aqueous phase content in each formulation. An increase in the aqueous phase beyond 30% leads to a marked increase in viscosity, reflecting the swelling behavior of PPG-5-CETETH-20 and the resulting transition from a fluid to a more structured system.^33^

**Figure 2:**
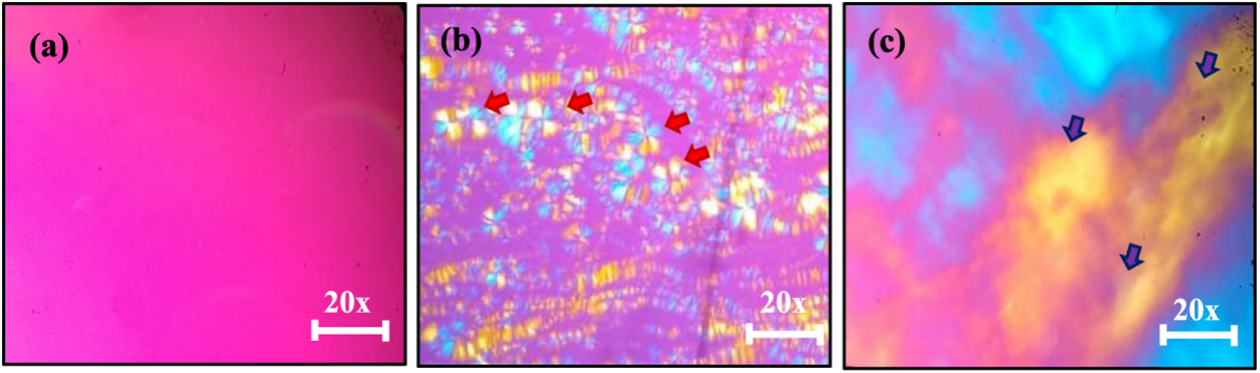
Description of Liquid-Crystalline (LC) mesophases: (a) Dark field, (b) Maltese-cross (red arrows), and (c) Hexagonal (purple arrows). Images were acquired at a magnification of 20× using an Olympus BX41 microscope fitted with a QColor3 camera (Olympus America Inc.).

The LC mesophases of formulations LC_5_, LC_6_, and LC_10_ were characterized using PLM, where a polarizer attached to the condenser directs the light beam in a single direction (Figure 2). LC samples capable of deviating the light beam are classified as anisotropic, whereas those that do not are termed isotropic.^34^ Lamellar and hexagonal mesophases exhibit anisotropic behavior, as they can deviate the light beam.^35^ PLM reveals specific optical textures for these mesophases, such as Maltese-cross formations and striations. In contrast, cubic structures and microemulsions are optically isotropic, do not interact with polarized light, and are visualized as dark areas.^36^

LC formulations can be classified based on their fluidity and viscosity properties. Microemulsions, for instance, are characterized as fluid and transparent formulations, whereas cubic liquid-crystalline mesophases appear viscous and transparent.^37,38^

In general, for the fluid formulations containing AgNPs, mesophases of the Maltese-cross type were identified, except for formulation LC_10_ (Figure 3 (g) and (h)), which exhibited a transition from a dark field to the lamellar phase, displaying extensive Maltese-cross regions after AgNPs incorporation. In formulation LC_6_, no significant mesophase changes were observed (Figure 3 (e) and (f)). However, the molecules arranged into a spaced network-like structure in the presence of AgNPs. Conversely, in the more viscous LC_5_ formulation, minimal alterations were detected in the hexagonal mesophases (Figure 3 (c) and (d)).

**Figure 3:**
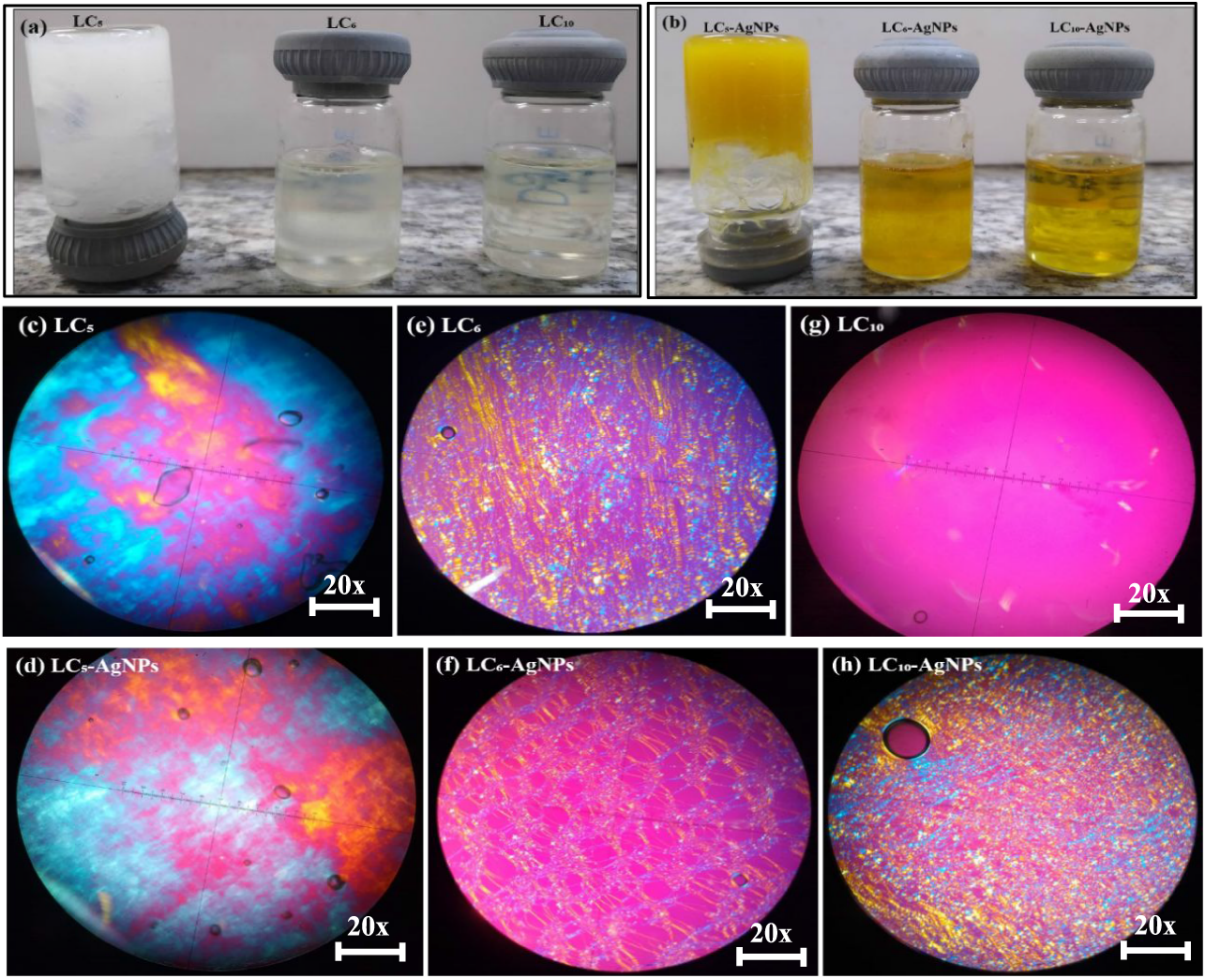
Image of Liquid-Crystalline (LC) formulations: (a) LC_5_, LC_6_, and LC_10_, and (b) LC_5_-AgNPs, LC_6_-AgNPs, and LC_10_-AgNPs. Photomicrographs of LC formulations: Polarized Light Microscopy (PLM) images of mesophases: (c) LC_5_: Hexagonal, and (d) LC_5_-AgNPs: Hexagonal; (e) LC_6_: Maltese-cross/Hexagonal, and (f) LC_6_-AgNPs: Maltese-cross/Hexagonal; (g) LC_10_: Dark Field, and (h) LC_10_-AgNPs: Maltese-cross/Hexagonal. The images were obtained at 20× magnification using an Olympus BX41 microscope equipped with a QColor3 camera (Olympus America Inc.).

These results underscore the importance of formulation composition and phase proportions in determining mesophases and the physicochemical properties of liquid-crystalline formulations.^39^ The presence of oleic acid and PPG-5-CETETH-20 was crucial in modulating rheological and structural characteristics, allowing for adjustments in fluidity and viscosity. Furthermore, the incorporation of AgNPs revealed specific interactions with these molecules that influenced the mesophases, leading to the formation of characteristic anisotropic structures, such as Maltese-crosses, and localized structural alterations, as observed in formulations LC_6_ and LC_10_.^40^

PLM is a valuable tool for the identification and characterization of liquid-crystalline mesophases, providing detailed information about molecular organization.^41^

### 3.2 Texture Profile Analysis

Mechanical characterization, encompassing hardness, compressibility, cohesiveness, and bioadhesiveness, is essential for the proper development of advanced pharmaceutical formulations. These measurements help determine the response of liquid-crystalline formulations to physiological stresses on the skin and provide valuable information on their structural features, which can influence clinical outcomes, as evidenced by earlier studies on topical applications involving oral and vaginal mucosa.^21,42^ Hardness is defined as the material’s resistance to deformation when a force is applied to a specific area.^43^ Compressibility, on the other hand, assesses a material’s capacity to undergo volume changes under applied pressure, which relates to its spreadability and ease of use. Bioadhesiveness refers to the ability of a formulation to adhere to biological surfaces, prolonging its residence duration at the application site. Lastly, cohesiveness indicates the material’s ability to resist separation and maintain its structure under different types of mechanical stress, including compression, ensuring its integrity during use.^44^

The results presented in Table 2 provide a detailed analysis of the mechanical behavior of the evaluated formulations.

**Table 2:** Mechanical Properties of the Developed Liquid-Crystalline Formulations: Hardness (N), Compressibility (N·s), Cohesiveness (−), and Bioadhesiveness (N·s)

Formulation	Hardness (N)	Compressibility (N·s)	Cohesiveness (−)	Bioadhesiveness (N·s)
LC_5_	1.677 ± 0.017	0.203 ± 0.020	5.468 ± 0.044	3.858 ± 0.044
LC_5_-AgNPs	1.887 ± 0.143	0.063 ± 0.007	5.671 ± 0.196	3.558 ± 0.278
LC_6_	0.659 ± 1.251	20.522 ± 0.181	1.319 ± 0.129	0.027 ± 0.056
LC_6_-AgNPs	0.168 ± 0.164	20.123 ± 0.056	0.050 ± 0.429	0.491± 0.260
LC_10_	0.610 ± 2.184	24.642 ± 6.752	2.446 ± 0.783	0.84 ± 0.262
LC_10_-AgNPs	0.250 ± 0.086	38.702 ± 13.511	2.083 ± 1.138	0.54 ± 0.135

According to the experimental data, the hardness values observed for the LC_5_ (1.677 ± 0.017 N) and LC_5_-AgNPs (1.887 ± 0.143 N) hexagonal mesophase semisolid formulations suggest stronger interactions between the matrix components, providing greater resistance to initial deformation. A reduced compressibility value is desirable to facilitate the removal of the gel from its container and ensure uniform application over the injured area.^45^ The low compressibility observed for the LC_5_ (0.203 ± 0.020 N·s) and LC_5_-AgNPs (0.063 ± 0.007 N·s) formulations indicates less deformation under pressure, which is an advantageous characteristic for *in situ* formulations that require structural stability after application at the target site. The cohesiveness values reflect the material’s ability to maintain structural integrity under mechanical stress, thereby minimizing the risk of disintegration during use.^46^ Lastly, the elevated bioadhesiveness confirms the material’s suitability for effective adhesion to biological surfaces, such as skin, ensuring prolonged retention at the application site.^47^ These properties make the formulations promising candidates for wound treatment and tissue regeneration.

**Figure 4:**
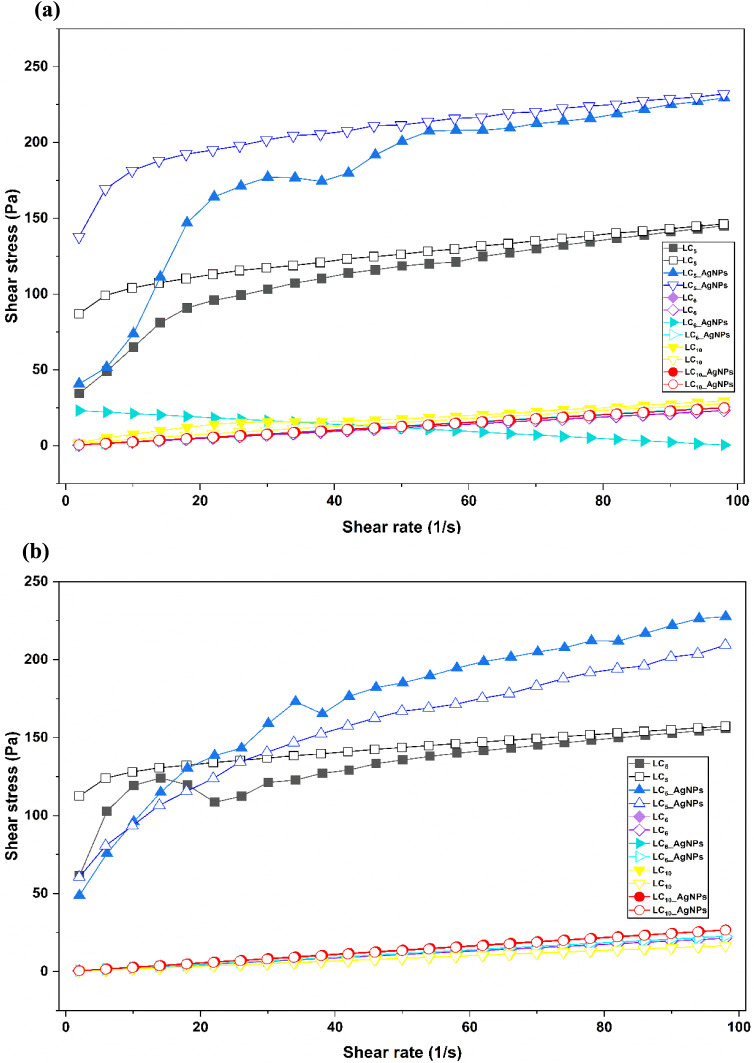
Flow rheograms of the formulations illustrating the correlation between shear stress and shear rate. Solid symbols indicate the ascending curves, whereas hollow symbols denote the descending curves. Temperature: 25 ± 0.5 °C (a)—LC_5_, LC_6_, LC_10_, LC_5_-AgNPs, LC_6_-AgNPs, and LC_10_-AgNPs; Temperature: 32 ± 0.5 °C (b)—LC_5_, LC_6_, LC_10_, LC_5_-AgNPs, LC_6_-AgNPs, and LC_10_-AgNPs. Results are expressed as the average of three independent measurements (*n* = 3) for each formulation. LC: liquid crystal; AgNPs: silver nanoparticles.

In contrast, the LC_6_, LC_6_-AgNPs, LC_10_, and LC_10_-AgNPs formulations exhibited lower mechanical resistance and higher compressibility, which can be attributed to their less structured consistency. The low bioadhesiveness values indicate a limited ability to adhere to biological surfaces, such as skin or mucosa. Nevertheless, these formulations may be more suitable for applications requiring high spreadability, rapid absorption, or frequent reapplication, depending on the stage of contamination and wound depth.

### 3.3 Rheological Studies

Continuous rheological measurements enable evaluation of a material’s flow behavior under different stress conditions, providing a direct link between its microstructure and macroscopic properties.^48^ This behavior is represented by a flow curve, which relates shear stress to shear rate, as illustrated in Figure 4. The curve consists of two distinct regions: the ascending segment, which indicates the material’s response to increasing shear rate, and the descending segment, which describes its behavior as the shear rate decreases.^49^

According to this model, fluids are categorized as dilatant when η exceeds 1 and as pseudoplastic when η is lower than 1. An η value of 1 indicates Newtonian flow behavior. Formulation viscosity is determined using the consistency index (*k*), a parameter that shows a direct relationship with the viscosity of the LC system. Values of η and k are summarized in Table 3.

**Table 3:** Rheological Flow Results Including the Flow Index (η), Consistency Index (*k*), and Linear Regression Coefficients (*R* ^2^), Obtained at Temperatures of 25 ± 0.5 °C and 32 ± 0.5 °C^a^

	Temperature (°C)					
	25 ± 0.5			32 ± 0.5		
Formulations	η	*k*	*R* ^2^	η	*k*	*R* ^2^
LC_5_	0.32	33.08	0.98	0.17	68.63	0.90
LC_5_-AgNPs	0.36	45.80	0.95	0.35	45.10	0.99
LC_6_	1.00	0.24	1.00	1.00	0.22	0.99
LC_6_-AgNPs	1.00	0.26	0.99	1.00	0.23	0.99
LC_10_	0.57	2.05	0.97	1.00	0.18	1.00
LC_10_-AgNPs	1.00	0.26	1.00	1.00	0.25	0.99

a These values were determined using TRIOS software from TA Instruments.

Based on the analysis of the ascending curve, materials can be classified as Newtonian or non-Newtonian. Newtonian materials maintain a constant viscosity independent of the applied shear rate, producing a linear flow curve that originates at zero.^50^ Conversely, non-Newtonian materials fall into three main categories: (i) pseudoplastic, which exhibit decreasing viscosity with increasing shear rate due to particle alignment in the direction of flow (shear thinning); (ii) plastic, which require a minimum stress (yield stress) to initiate flow but behave similarly to pseudoplastic materials once this threshold is exceeded; and (iii) dilatant, which display progressively increasing viscosity as shear rate rises.^48^

The descending curve provides further insights into a material’s structural recovery capacity, enabling classification as thixotropic or antithixotropic. Thixotropic materials exhibit a decrease in viscosity under shear, returning to their initial state upon stress removal. This behavior may be time-dependent or time-independent and is often represented graphically by a hysteresis loop.^51^ Conversely, antithixotropic (rheopectic) materials exhibit increasing viscosity under shear, with structural recovery occurring after stress ceases. In these cases, the descending curve appears above the ascending curve, following a counterclockwise direction.^50^

Based on the values of the η, it was observed that the LC_5_, LC_5_-AgNPs, and LC_10_ formulations exhibited pseudoplastic behavior (η < 1) at 25 °C, associated with lower viscosity values at higher shear rates. This effect was more pronounced in the LC_5_ (η = 0.32; *k* = 33.08) and LC_5_-AgNPs (η = 0.36; *k* = 45.80) formulations, which displayed the lowest η values at this temperature. Incorporating AgNPs into LC_5_ resulted in a modest rise in viscosity, as indicated by the k value, suggesting a structural modification of the LC system. The LC_10_ formulation (η = 0.57; *k* = 2.05) also showed pseudoplastic behavior, though to a lesser extent. Furthermore, LC_5_ and LC_5_-AgNPs exhibited negative hysteresis areas in the rheological curves, indicating rheopectic behavior—i.e., an increase in viscosity over time under shear stress, as discussed in the results. At 32 °C, most formulations exhibited Newtonian behavior (η = 1), except for LC_5_ (η = 0.17; *k* = 68.63) and LC_5_-AgNPs (η = 0.35; *k* = 45.10), which retained their pseudoplastic profiles, demonstrating resistance to complete shear thinning even under simulated body temperature. The higher k value for LC_5_ at this temperature suggests a significant increase in viscosity compared to 25 °C, possibly due to a denser structural reorganization. LC_5_-AgNPs maintained similar viscosity values (*k*) but showed less sensitivity to temperature changes, indicating greater structural stability. This formulation also exhibited pseudoplastic–thixotropic characteristics, with time-dependent shear thinning, which is advantageous for topical application, ensuring good spreadability and moderate structural recovery after shear. The formulations LC_6_ (*k* = 0.24 at 25 °C and 0.22 at 32 °C), LC_6_-AgNPs (*k* = 0.26 and 0.23, respectively), LC_10_-AgNPs (*k* = 0.26 and 0.25), and LC_10_ at 32 °C (*k* = 0.18) exhibited η values equal to 1, indicating Newtonian behavior at both tested temperatures. The corresponding *k* values reflect relatively low and stable viscosities, typical of systems with linear responses to increasing shear rate.

Therefore, considering the updated rheological data, the LC_5_-AgNPs formulation stands out as the most promising candidate for the treatment of wounds requiring controlled release, due to its combination of pseudoplastic–rheopectic behavior. This versatility supports both localized application and prolonged retention at the wound site, promoting more sustained release of AgNPs. In contrast, formulations with Newtonian behavior and low viscosity, such as LC_6_, LC_6_-AgNPs, and LC_10_-AgNPs, are more suitable for less critical wounds or for covering larger areas, where good spreadability and ease of application are desirable. Hence, the rheological profiles of the evaluated formulations may be strategically selected according to the complexity and therapeutic needs of the wound.

#### 3.3.1 Oscillatory Rheological Analysis

The oscillatory analysis was conducted under two distinct thermal conditions: at 25 ± 0.5 °C, aiming to evaluate the viscoelastic response of the formulations at ambient temperature, and at 32 ± 0.5 °C, which represents skin surface conditions, which are more representative of the physiological cutaneous environment. The results are reported in Table 4.

**Table 4:** Oscillatory Rheology Results Expressed as Apparent Viscosity (η) and Regression Coefficients (*R* ^2^), Determined at Temperatures of 25 ± 0.5 °C and 32 ± 0.5 °C^a^

Temperature (°C)						
	25 ± 0.5			32 ± 0.5		
Formulations	η	*R* ^2^	*G*′/*G*″	η	*R* ^2^	*G*′/*G*″
**LC**_**5**_	0.15	0.90	5.08	0.24	0.96	3.17
**LC**_**5**_ **-AgNPs**	0.12	0.94	5.75	0.06	0.97	7.32
**LC**_**6**_	1.17	0.99	0.22	1.65	0.92	0.011
**LC**_**6**_ **-AgNPs**	1.17	0.92	0.05	1.87	0.96	0.007
**LC**_**10**_	2.06	0.91	0.002	1.77	0.94	0.007
**LC**_**10**_ **-AgNPs**	0.96	1.80	0.006	2.56	0.99	0.007

a Data analysis was performed using TRIOS software (TA Instruments).

Under the 25 °C condition, as shown in Figure 5(a), the LC_5_ (η = 0.15; *R* ^2^ = 0.90) and LC_5_-AgNPs (η = 0.12; *R* ^2^ = 0.94) formulations exhibited predominantly elastic behavior (*G*′ > *G*″), indicating the presence of a partially organized three-dimensional structure, typical of gel-like systems. This profile suggests greater resistance to deformation and improved structural recovery capacity following stress relief—desirable properties for topical formulations requiring bioadhesion and sustained release of active agents, such as AgNPs.^52^ In contrast, the LC_6_ (η = 1.17; *R* ^2^ = 0.99), LC_6_-AgNPs (η = 1.17; *R* ^2^ = 0.92), LC_10_ (η = 2.06; *R* ^2^ = 0.91), and LC_10_-AgNPs (η = 0.96; *R* ^2^ = 1.80) formulations displayed predominantly viscous behavior (*G*″ > *G*′), associated with a less organized internal structure and greater energy dissipation. The high apparent viscosity observed for LC_10_ (η = 2.06) confirms its more fluid-like character. Although the incorporation of AgNPs into the LC_10_ formulation reduced its viscosity, the loss modulus (*G*″) remained higher than the storage modulus, maintaining the system’s viscous profile.

**Figure 5:**
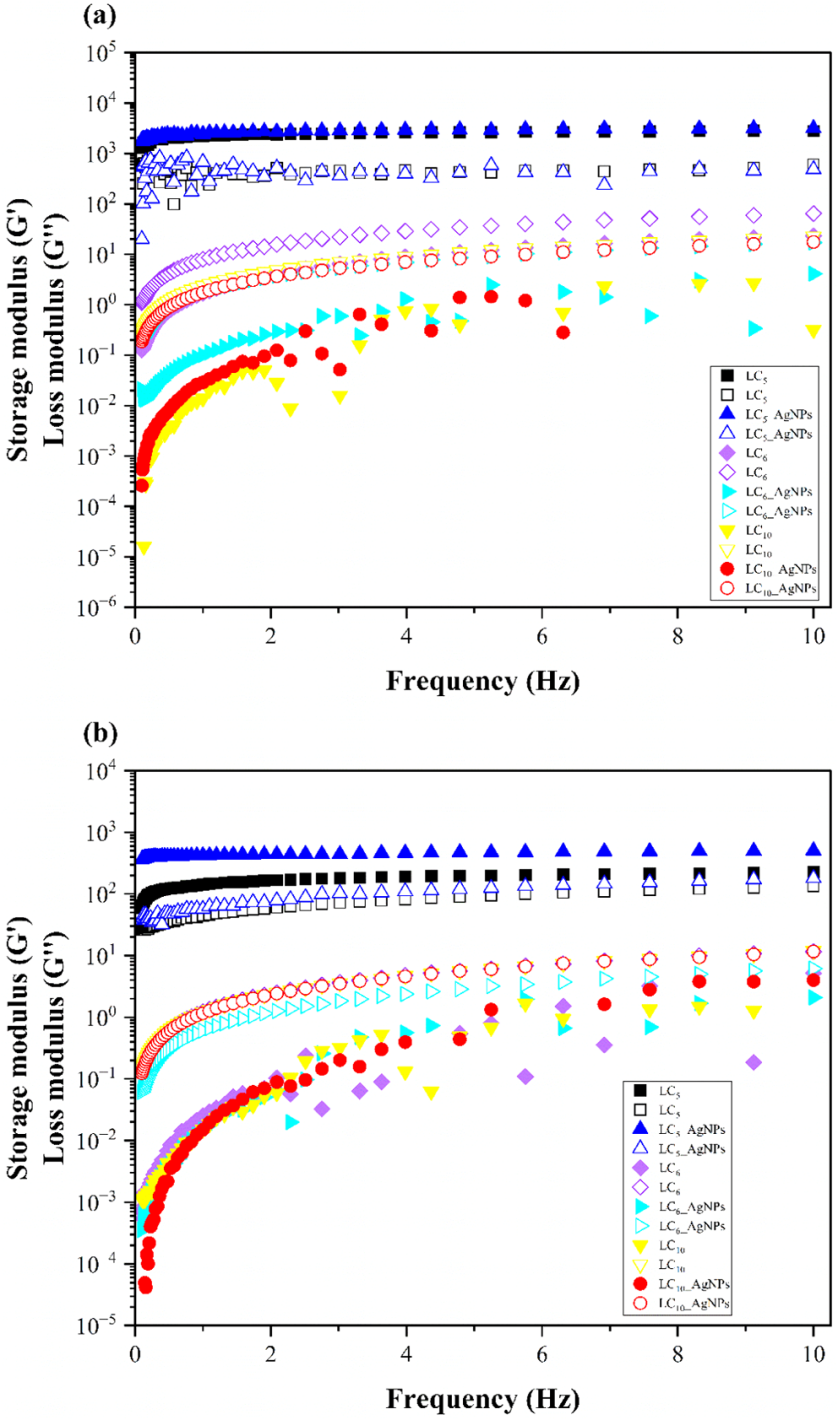
Storage modulus (*G*′) and loss modulus (*G*″) as a function of frequency (Hz) for the LCs formulations. Solid symbols indicate the ascending curves, whereas hollow symbols denote the descending curves. (a) Measurements performed at 25 ± 0.5 °C: LC_5_, LC_6_, LC_10_, LC_5_-AgNPs, LC_6_-AgNPs, and LC_10_-AgNPs. (b) Measurements performed at 32 ± 0.5 °C: LC_5_, LC_6_, LC_10_, LC_5_-AgNPs, LC_6_-AgNPs, and LC_10_-AgNPs. Results are expressed as the average of three independent measurements (*n* = 3) for each formulation. LC: liquid crystal; AgNPs: silver nanoparticles.

At a standardized experimental temperature of 32 ± 0.5 °C, as shown in Figure 5 (b), the LC_5_ and LC_5_-AgNPs formulations maintained predominantly elastic behavior, with *G*′ > *G*″ and viscosities of 0.24 (*R* ^2^ = 0.96) and 0.06 (*R* ^2^ = 0.97), respectively. This reinforces the presence of structured networks capable of providing mechanical resistance and suitability for topical application, even under body temperature. The other formulations, in turn, exhibited viscous behavior, with *G*″ > *G*′ and viscosities ranging from 1.65 to 2.56 (*R* ^2^ between 0.92 and 0.99), reflecting higher fluidity and lower structural cohesion.

These results highlight the distinct rheological behaviors among the LC formulations and emphasize the importance of oscillatory testing as a robust tool for characterizing complex viscoelastic systems. The comparison between the analyses at 25 °C and 32 °C allows for the observation of thermal stability or sensitivity of the systems, aiding in the selection of the most suitable formulation for topical application in cutaneous wounds. Among the evaluated systems, the LC_5_ and LC_5_-AgNPs formulations stand out as the most promising for the treatment of critical wounds, as they combine gel-like behavior, bioadhesive properties, and the potential for controlled release of AgNPs—features that are desirable for ensuring prolonged contact with the wound bed, supporting tissue repair while minimizing the necessity for repeated applications. On the other hand, the remaining formulations, which exhibited viscous-dominant profiles, are more appropriate for application on superficial lesions, where high spreadability and low resistance to deformation are preferred characteristics, facilitating application and uniform coverage of the injured area.

### 3.4 *In Vitro* and *Ex Vivo* Release Studies of LC-AgNPs Formulations

The release profiles of AgNPs loaded into LC formulations were investigated by measuring Ag concentrations using ICP-MS. The release curves, shown in Figure 6, illustrate the percentage of AgNPs released in both *in vitro* and *ex vivo* studies, as depicted in Figures (a) and (b), respectively.

**Figure 6:**
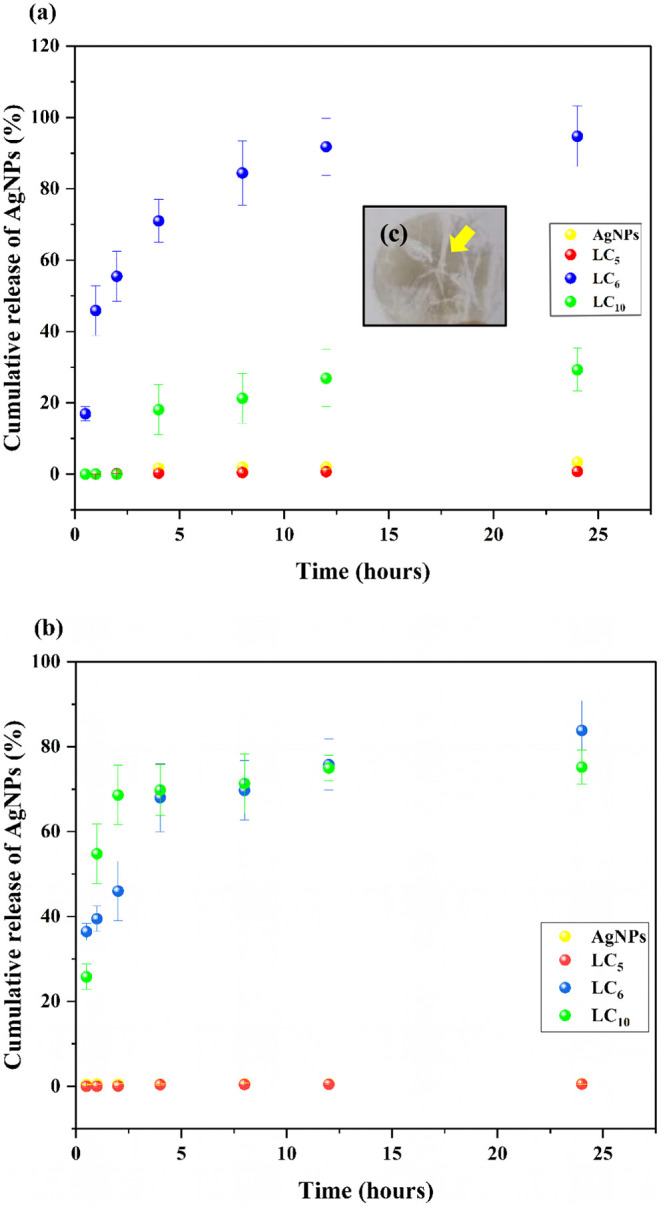
Cumulative release profile of silver nanoparticles (AgNPs) from lyotropic liquid-crystalline formulations: (a) *In vitro* release profile (% cumulative release) as a function of time (h); (b) *Ex vivo* permeation profile through pig ear skin; (c) Representative image showing AgNPs precipitated on a cellulose acetate membrane after diffusion assay.

The *in vitro* release profile of AgNPs (23,4 μg/mL) demonstrated a rapid release within the first 4 h, with approximately 2.0% (0.46 μg/mL) of AgNPs released, reaching 3.0% (0.702 μg/mL) after 24 h. The AgNPs permeation assays revealed a release of 0.47% (0.109 μg/mL) within the first 30 min, 0.6% (0.140 μg/mL) after 1 h, followed by a steady release of approximately 0.5% (0.117 μg/mL) from 2 h until the end of the experiment (24 h) (Figure 6 (a)). This release phenomenon is likely attributed to the oxidation of silver, as observed through visual assessment (Figure 6 (c)), with the formation of grayish precipitates overlying the cellulose acetate membrane used in the Franz cells. The instability was attributed to the acidic environment (pH = 4.0) of the simulated exudate, which contains soluble sodium and calcium ions. These conditions disrupted the balance of reducing agents adsorbed on the nanoparticle surfaces, thermodynamically favoring silver aggregation. This process led to an increase in nanoparticle diameter and the blockage of cellulose acetate membrane pores, ultimately halting the diffusion process.^53^ The aggregation state of nanoparticles is closely linked to their reactivity within biological compartments. Factors such as surface chemistry, pH, and ionic strength influence both the propensity of AgNPs to aggregate and their size. The excess protons in acidic environments promote the protonation of citrate anions, leading to the loss of negative charges and facilitating nanoparticle aggregation.^54^ These findings are in agreement with those described by Sharma et al., who observed the formation of aggregates in sodium citrate-coated AgNPs under acidic conditions.^55^ The variation in pH is particularly relevant to wound pathophysiology. Chronic wounds exhibit a direct relationship between alkalinity and chronicity (pH > 7.0). Conversely, under alkaline conditions, AgNPs maintain structural stability, suggesting their suitability for antimicrobial therapies targeting infected wounds in more advanced chronic stages.^56^ In any nanocolloidal suspension of AgNPs, chemical equilibrium is established by the AgNPs themselves, free silver, and adsorbed silver on their surfaces.^57^ To address the limitation of low concentrations of released AgNPs, controlled release systems present an effective solution to maintain AgNP’ stability under biological conditions.^58^ In studies conducted by Kraeling et al., the *in vitro* percutaneous penetration of aqueous AgNPs in porcine skin was evaluated.^59^ The key finding was that most AgNPs were not absorbed by the skin in an *in vitro* diffusion system after 24 h, remaining primarily in the stratum corneum with minimal penetration into the dermis. Similarly, our results revealed very low but detectable amounts of silver in the stratum corneum and dermis (0.5%) after 24 h. The low permeability of aqueous AgNPs is attributed to the lipophilic nature of the skin layers.^60^ *In vitro* release and *ex vivo* permeation assays revealed distinct behaviors of AgNPs (reduced and aggregated states) depending on pH, as well as their limited permeation due to the skin’s lipophilic properties. These results offer important insights into the practical use of AgNPs in medical applications.

The incorporation of AgNPs into LC-based formulations offered protection against oxidative processes.^61^ The structural organization of liquid crystals—composed of PPG-5-CETETH-20, oleic acid, and aqueous AgNPs suspension—creates a microstructured environment that limits direct interaction between nanoparticles and oxidizing agents. This system acts as both a physical and chemical barrier, reducing AgNP’ exposure to unfavorable environmental conditions such as pH variations and the presence of oxidizing ions.^62^ Among the evaluated LC formulations, the hexagonal mesophase LC_5_ exhibited the lowest AgNPs release rates, ranging from 0.5% (0.07 μg/mL) to 1.0% (0.14 μg/mL) (*p* < 0.001) over 24 h. In contrast, formulations with lamellar and hexagonal mesophases, LC_6_ and LC_10_, released approximately 90% (4.21 μg/mL) and 25% (1.17 μg/mL) of AgNPs, respectively, in the *in vitro* model (Figure 6 (a)). Notably, LC_10_ (Figure 6 (b)) showed a significant increase to 50% (2.34 μg/mL) release in the *ex vivo* model over the same time period. The varying percentages of AgNPs released among the liquid-crystalline formulations are directly linked to the distinct mesophases evaluated: lamellar, hexagonal, and cubic.^17^ These mesophases exhibit unique structural characteristics that influence the release rates of active agents, primarily through diffusion mechanisms and, to a lesser extent, matrix erosion.^63^ Lamellar phases consist of alternating layers of surfactant molecules and water, creating planar structures that modulate diffusion pathways.^64^ Hexagonal phases are characterized by cylindrical arrangements of surfactant molecules, forming channels that facilitate or hinder diffusion depending on their dimensions and interactions with encapsulated agents. Cubic phases possess a three-dimensional network of interconnected channels, offering a more tortuous path for diffusion, which may result in slower release rates.^65^ The dielectric constants within these mesophases vary due to differing oil and water region compositions, further affecting the behavior of encapsulated substances. These structural nuances are crucial when designing delivery systems for pharmaceuticals and other active compounds, as they enable tuning of release profiles to achieve desired therapeutic outcomes.^66^ Lamellar mesophases, observed in LC_6_ and LC_10_ formulations, exhibited higher AgNPs release rates compared to the hexagonal LC_5_ formulation. This behavior can be attributed to the less cross-linked lamellar network, which facilitates molecular transport and results in diffusion rates similar to those observed in AgNPs-free suspensions.

The rheological and textural data support these observations. The LC_6_ and LC_10_ formulations exhibited greater fluidity and lower cohesiveness—typical characteristics of lamellar structures—which favor higher release rates of active agents. In contrast, the LC_5_ formulation, with its more organized and densely interwoven hexagonal structure, demonstrated greater mechanical resistance, contributing to a more controlled and prolonged release of AgNPs. These results highlight the influence of the structural and mechanical properties of liquid-crystalline mesophases on the release profiles of encapsulated active agents. Release assays using porcine stratum corneum revealed significant differences compared to the cellulose acetate membrane model, emphasizing the biological substrate’s impact. While the LC_10_ formulation released 20% in the first hours in the cellulose acetate model, it released over 70% in the porcine skin model during the same time frame, maintaining this release rate until the experiment’s conclusion. This behavior can be attributed to the lipid composition and structure of the stratum corneum, which favor interaction with the LC_10_ formulation—comprising 50% surfactant (PPG-5-CETETH-20), 30% oil phase (oleic acid), and 20% aqueous AgNPs suspension—and the skin barrier. The presence of lipid microdomains in the stratum corneum can act as reservoirs for active agents, contributing to the observed increase in release.^14^ In contrast, the LC_5_ formulation released between 0.5% and 1.0% of AgNPs in both the cellulose acetate model and the *ex vivo* assay, with results resembling those observed for AgNPs-free suspensions. The LC_6_ formulation exhibited a similar release profile in both models. In the cellulose acetate model, it released over 40% (1.87 μg/mL) within the first few hours, nearly reaching the total 4.68 μg/mL by the end of the experiment. In the stratum corneum assay, over 60% (2.80 μg/mL) was released within the first 5 h.

Using porcine skin in AgNPs release assays is particularly relevant for wound treatments, as porcine skin exhibits structural and functional similarities to human skin, including the presence of the stratum corneum. This model simulates the physiological conditions of wounds, providing more accurate and clinically applicable data.^67^

Formulations with liquid-crystalline mesophases, such as maltese-cross and hexagonal, release AgNPs more rapidly, making them suitable for immediate antibacterial action in acute wounds. In contrast, the LC_5_ formulation, characterized by a slower and more controlled release profile, is more appropriate for chronic wounds. In summary, the results underscore the significance of controlled release systems tailored to specific wound conditions and highlight the importance of using relevant biological models, such as porcine skin, to enhance the therapeutic application of AgNPs in wound treatments and complex wounds.

#### 3.4.1 Mathematical Models

Bioactive release from drug delivery systems is a complex process often described using mathematical kinetic models. In this study, several models—including Baker & Lonsdale, Korsmeyer–Peppas, Hixson–Crowell, Higuchi, and first-order—were tested, with goodness of fit assessed via the regression coefficient (*R* ^2^) (Table 5). The Korsmeyer–Peppas model proved most suitable for characterizing AgNP release from the LC systems, except for the LC_6_ formulation in vitro. This semiempirical model relates drug release to time through an exponential equation, with the release exponent *n* indicating the predominant mechanism governing the process. When *n* ≤ 0.45, the release is primarily diffusion-controlled, characterizing a predominantly Fickian process, where the release rate is governed by drug diffusion through the medium. If *n* lies between 0.45 ≤ *n* ≤ 0.89, the release is considered non-Fickian, indicating a multifactorial process involving not only drug diffusion but also mechanisms such as dissolution and matrix erosion. Finally, when *n* ≥ 0.89, the release is dominated by mixed mechanisms such as sorption, stress, and polymer chain rupture, reflecting a more complex release process that is highly dependent on environmental conditions. These values and their associated mechanisms are critical for optimizing delivery systems, allowing for precise modulation of release rates to meet specific therapeutic needs.^68,69^

**Table 5:** Experimental Correlation Coefficients (*R* ^2^) Obtained from the Mathematical Models Applied to the Liquid Crystal (LC) Formulations^a^

	Mathematical Models					
	Adjusted *R* ^2^ values					
Formulations	Assay	Baker and Lonsdale	Korsmeyer–Peppas	Hixon and Crowell	Higuchi	First-order
AgNPs-free	*in vitro*	-	0.98	-	-	-
			*n* = 0.037			
	*ex vivo*	0.98	0.98	0.93	0.97	0.93
			*n* = 0.57			
LCM_5_-AgNPs	*in vitro*	0.96	0.97	0.87	0.96	0.87
			*n* = 0.58			
	*ex vivo*	0.94	0.94	0.83	0.94	0.83
			*n* = 0.54			
LC_6_-AgNPs	*in vitro*	0.98	0.97	0.95	0.95	0.98
			*n* = 0.40			
	*ex vivo*	0.91	0.98	-	0.64	0.58
			*n* = 0.25			
LC_10_-AgNPs	*in vitro*	0.98	0.99	0.98	0.98	0.98
			*n* = 0.68			
	*ex vivo*	0.72	0.90	-	0.49	-
			*n* = 0.23			

a Statistical analyses were performed using SigmaPlot software.

In applying the Korsmeyer–Peppas model, the results indicated that the data fitting for the AgNPs-free samples (*in vitro*), LC_6_ (*ex vivo*), and LC_10_ (*ex vivo*) demonstrated a diffusion-controlled release behavior, with *n* values <0.45. These values suggest a pseudo-Fickian process, where the release curves resemble Fickian diffusion but reach equilibrium more rapidly. The significantly low *n* value observed for AgNPs (*n* = 0.037) indicates an extremely limited diffusion capacity, likely associated with nanoparticle aggregation.

Conversely, non-Fickian behavior, characterized by *n* values between 0.45 and 0.89, was observed for the AgNPs-free samples (*ex vivo*), LC_5_ (*in vitro* and *ex vivo*), and LC_10_ (*in vitro*). This anomalous transport results from a combination of Fickian diffusion and Case II transport mechanisms, the latter being controlled by the transition of polymer chains from a semirigid to a more flexible state. These processes involve swelling and chain relaxation, as described by Ritger and Peppas (1987).^70^

The release of AgNPs, when incorporated into liquid-crystalline delivery systems, occurs in a controlled and sustained manner, governed by swelling and diffusion mechanisms. Upon contact with simulated exudate fluid, the hydrophilic portion of the liquid crystal’s swells, opening aqueous channels and enabling the gradual release of AgNPs according to Fickian transport laws.

The use of mathematical models is essential for understanding and predicting the release profiles of active agents in drug delivery systems. Models such as Korsmeyer-Peppas enable the identification of the predominant release mechanism, whether Fickian or non-Fickian, providing critical data for effective and optimized formulation development.

### 3.5 Antimicrobial Assays

LC-based systems containing bioactive molecules and AgNPs show promising antimicrobial potential.^71–73^ In this study, their activity was assessed via the agar diffusion method against *S. aureus* (ATCC 25923), *P. aeruginosa* (ATCC 27853), and *E. coli* (ATCC 25922), with inhibition zone diameters reported as mean ± SD from three independent experiments (Table 6). Consistently, microdilution assays previously reported by Baveloni et al. (2024) showed MIC/MBC values of 6.74/13.5 μg/mL for *S. aureus* and *P. aeruginosa*, and 58.5/117 μg/mL for *E. coli*.^9^

**Table 6:** Agar Diffusion Antimicrobial Assay^a^

	Mean and standard deviation (SD) (mm)		
Samples	*S. aureus*	*P. aeruginosa*	*E. coli*
Ampicillin	9.0 ± 1.4	_	_
AgNPs	11.0 ± 1.3	11.00 ± 1.3	11.0 ± 1.3
LC_5_	_	_	_
LC_5_-AgNPs	14.0 ± 0.7	12.0 ± 0.5	13.0 ± 1.7
LC_6_	8.0 ± 1.0	9.0 ± 0.0	_
LC_6_-AgNPs	10.0 ± 0.7	12.0 ± 0.3	5.0 ± 0.2
LC_10_	10.0 ± 0.3	15.0 ± 2.0	11.0 ± 4.0
LC_10_-AgNPs	11.0 ± 0.5	15.0 ± 2.0	12.0 ± 6.0

a Inhibition zone results for ampicillin, AgNPs (silver nanoparticles), and LC (liquid crystal) formulations against *S. aureus*, *P. aeruginosa*, and *E. coli*. Data are expressed as mean ± standard deviation and were processed using Microsoft Office Excel.

Ampicillin showed no antimicrobial efficacy against the Gram-negative strains *P. aeruginosa* and *E. coli*, as evidenced by the absence of inhibition zones, suggesting bacterial resistance mechanisms. This resistance is primarily associated with the production of β-lactamases, enzymes capable of inactivating the antibiotic, as well as modifications in penicillin-binding proteins (PBPs), which compromise its effectiveness.^74^ Evidence of this resistance was also observed in a study by Sivanmaliappan and Sevanan (2011), which analyzed 18 isolates of *P. aeruginosa* collected from diabetic foot ulcers in Coimbatore, India (June 2006–April 2007), confirming the ineffectiveness of ampicillin against these strains.^75^ Supporting these findings, a more recent investigation by Mączyńska et al. (2023) tracked antimicrobial resistance in Gram-negative *Enterobacterales*, such as *E. coli* and *Klebsiella pneumoniae*, isolated from patients at a multidisciplinary hospital in Wroclaw, Poland (2017–2021). The data revealed high rates of ampicillin resistance among the isolates, as demonstrated by both disk diffusion and enzymatic detection methods.^76^

Among the tested formulations, LC_5_-AgNPs exhibited the highest efficacy in inhibiting bacterial growth. This antimicrobial activity is attributed to its higher concentration of AgNPs (60%, equivalent to 70.2 μg/mL), whereas LC_6_ and LC_10_, which contained 20% AgNPs (23.4 μg/mL), showed reduced effectiveness. Notably, the LC_5_ formulation without AgNPs failed to inhibit bacterial growth, underscoring the essential role of AgNPs in antimicrobial activity. Interestingly, the LC_6_ and LC_10_ formulations—even without AgNPs—demonstrated mild antimicrobial activity. This effect may be due to their higher proportion of oil phase, composed of oleic acid and PPG-5-CETETH-20, which contributed to bacterial growth inhibition. Oleic acid has been described as a bactericidal agent against *S. aureus* *in vitro *.^76–78^ Meanwhile, PPG-5-CETETH-20, as a nonionic surfactant, facilitates the interaction between lipophilic components and bacterial membranes, thereby enhancing the antimicrobial efficacy of the system.

Liquid-crystalline formulations promoted the diffusion of AgNPs, whose antimicrobial action occurs predominantly through direct contact with bacterial cells. Evidence for this mechanism was provided by Singh and Mijakovic (2022), who tested green-synthesized AgNPs (5–30 nm) against *E. coli* and *P. aeruginosa*.^77^ Through electron microscopy, the authors observed that the particles adhere to bacterial surfaces, penetrate the cell membrane, induce cytoplasmic retraction, and ultimately rupture the cell wall—demonstrating a direct mechanical action on the membrane. The developed system enabled sustained release of silver nanoparticles over time, significantly enhancing antimicrobial efficacy against the tested strains. Results confirmed AgNPs release from LC systems with a hexagonal mesophase (LC_5_ and LC_5_-AgNPs), as well as from systems with maltese-cross or lamellar/hexagonal organization (LC_6_, LC_6_-AgNPs, LC_10_, and LC_10_-AgNPs). This aligns with Kazeminava et al. (2021), who reported that PEG-based hydrogels containing AgNPs produced significantly larger inhibition zones (∼15 mm) compared with free AgNPs (∼10 mm), highlighting the contribution of the delivery system for sustained release and enhanced antimicrobial activity. The authors observed relevant antibacterial effects against *S. aureus* ATCC 25923 and *E. coli* ATCC 25922, with inhibition zones ranging from 10 to 18 mm for the nanocomposite hydrogels.^78^ Moreover, samples with higher AgNPs concentrations exhibited greater antibacterial toxicity, reinforcing the influence of nanoparticle load on antimicrobial performance.

Overall, the data demonstrate the potential of liquid-crystalline systems as efficient platforms for incorporating and controlling the release of AgNPs, thereby optimizing their antimicrobial activity against various bacterial strains. The LC_5_-AgNPs formulation stood out as the most effective, owing to its high AgNPs concentration and the organized structure of the hexagonal mesophase, which facilitated nanoparticle diffusion and direct contact with bacterial cells. Additionally, the synergistic contribution of excipients with antimicrobial properties, such as oleic acid, highlights the critical role of formulation composition in determining therapeutic performance.^79,80^ These findings align with previous studies on other delivery systems, demonstrating that immobilization and modulated release of AgNPs can significantly increase efficacy against resistant microorganisms. Therefore, the developed formulations represent promising candidates for the creation of new topical antimicrobial devices, particularly in clinical settings facing multidrug-resistant strains.

### 3.6 Evaluation of the Cytotoxicity of LC Formulations

The cytocompatibility of the LC formulations was examined using a qualitative agar diffusion method with L-929 murine fibroblasts as the test model. The results, expressed in millimeters of inhibition halo (mean ± standard deviation), allowed for the classification of the cytotoxicity levels of the tested samples based on the extent of the halo observed, as shown in Table 7.

**Table 7:** Cytotoxicity Evaluation in L929 Cells Showing Inhibition Halo Diameters for LC Formulations^a^

L929		
LC Formulations	Mean and SD (mm)	Toxicity
positive control	12.0 ± 1.0	Severe
AgNPs	-	Slight
LC_5_	9.0 ± 1.0	Moderate
LC_5_-AgNPs	11.0 ± 1.0	Severe
LC_6_	-	Slight
LC_6_-AgNPs	-	Slight
LC_10_	2.0 ± 1.0	Mild
LC_10_-AgNPs	3.0 ± 1.0	Mild

a Triton X-100 was used as the positive control. Samples included AgNPs, LC_5_, LC_5_-AgNPs, LC_6_, LC_6_-AgNPs, LC_10_, and LC_10_-AgNPs. Results are reported as mean ± standard deviation (mm) and were analyzed using Microsoft Office Excel.

The positive control exhibited a halo of 1.2 ± 0.1 mm and was classified as highly cytotoxic (severe toxicity), confirming the sensitivity of the method employed. In contrast, the isolated AgNPs suspension did not produce any inhibition halo, being classified as slightly cytotoxic in this assay. This behavior may be attributed to the limited diffusion of the nanoparticles through the agar matrix or to restricted indirect interaction with the cells—typical characteristics of the indirect contact methodology used. However, previous studies employing the same L929 cell line and the microdilution technique—which allows for direct and uniform contact between the particles and the cell monolayer—have reported significant cytotoxic effects associated with AgNPs.^9^ These effects have been widely linked to the induction of oxidative stress mediated by the generation of reactive oxygen species (ROS), ultimately leading to irreversible cellular damage.^81,82^

Among the formulations tested, LC_5_ produced a halo of 0.9 ± 0.1 mm and was classified as moderately cytotoxic. The LC_5_-AgNPs formulation, with a halo of 1.1 ± 0.1 mm, was classified as severely cytotoxic, showing a profile similar to the positive control. These findings suggest that the incorporation of AgNPs intensified the cytotoxicity of the formulation, possibly due to the higher aqueous fraction, which promotes nanoparticle release and diffusion. Although LC_5_-AgNPs exhibited strong antimicrobial efficacy, as previously discussed, its detrimental effects on fibroblasts warrant caution regarding its clinical use, particularly in wounds with impaired tissue regeneration. In contrast, LC_6_ and LC_6_-AgNPs did not form any inhibition halo and were classified as slightly cytotoxic. LC_10_ and LC_10_-AgNPs showed small halos of 0.2 ± 0.1 mm and 0.3 ± 0.1 mm, respectively, indicating mild to moderate cytotoxicity. Despite exhibiting lower antimicrobial activity, the formulations with higher lipid content showed a more favorable biocompatibility profile, which may support their application in continuous therapies or in less-infected wounds.

Collectively, the findings indicate that achieving a balance between the antimicrobial efficacy and cellular safety of AgNPs is highly dependent on the structural composition of the formulations, particularly their aqueous content. These results highlight the critical importance of selecting an appropriate liquid-crystalline mesophase to ensure safe and effective topical applications.

## 4 Conclusions

LC formulations proved to be promising systems for the sustained release of AgNPs, maintaining their stability against oxidation. The hexagonal mesophases exhibited more robust intermolecular interactions, with a gel-like structure and pseudoplastic–rheopectic behavior, promoting a slower and more controlled release profile. These characteristics make them more suitable for the treatment of wounds with higher exudation, which require a prolonged *in situ* release of AgNPs, that is, directly at the wound bed. In contrast, the lamellar mesophase formulations displayed a faster release profile and, according to rheological data, are more appropriate for superficial wounds with low contamination. Antimicrobial activity was more pronounced in formulations containing higher concentrations of AgNPs, whereas biocompatibility showed an inverse relationship, being negatively affected by increasing concentrations of these nanoparticles. These results support the potential of LCs as versatile and safe topical platforms for the treatment of bacterial skin infections, with the possibility of being tailored to specific clinical needs.
